# Advancing hospital-based health technology and impact assessments: institutionalizing strategic decision-making in innovation adoption

**DOI:** 10.1017/S026646232510336X

**Published:** 2026-01-19

**Authors:** Hao Yi Tan, Joshua Kuan Tan, Xiaohui Xin, Ruban Poopalalingam, Kenneth Kwek, Julian Thumboo, Mariam Krikorian Atkinson

**Affiliations:** 1 Health Services Research Unit, Singapore General Hospital, Singapore; 2 National Preventive Medicine Residency, National University Health System, Singapore; 3 Health Systems Group, Harvard School of Public Health, USA; 4 Medical Board, Singapore General Hospital, Singapore; 5 Chief Executive Officer, Singapore General Hospital, Singapore

**Keywords:** hospital-based health technology assessment (HB-HTA), innovation governance, strategic decision-making, health technology adoption, programme and impact evaluation, appropriate and value based healthcare

## Abstract

**Objectives:**

Hospitals are at the front line of adopting new health technologies, yet decision-making is often hampered by insufficient evidence, limited context, and misaligned priorities. Hospital-based health technology assessment (HB-HTA) provides a structured mechanism to evaluate innovations at the institutional level. However, published accounts of HB-HTA implementation remain scarce, particularly in Asian contexts.

**Methods:**

We describe Singapore General Hospital’s (SGH) approach to institutionalizing HB-HTA through a three-pronged strategy: (1) structured two-page narrative proposals to ensure clarity, rigor, and alignment with organizational priorities; (2) establishment of a dedicated HB-HTA team – the Impact Assessment, Program Evaluation, and Implementation Research team; and (3) targeted training programs to build capacity among clinical and managerial staff. Evaluation of the framework included formative feedback from proposers and senior leaders, surveys of participant satisfaction, and qualitative interviews.

**Results:**

Between 2023 and 2024, SGH piloted and scaled the HB-HTA framework, reviewing 15 proposals exceeding USD $40 million across domains, including artificial intelligence, robotics, diagnostics, and therapeutic devices. Training workshops engaged 55 participants with high Net Promoter Scores (≥50 percent), while surveys of senior leadership showed that 91 percent were satisfied or very satisfied with the process. Qualitative feedback highlighted improved strategic alignment, transparency, and confidence in decision-making.

**Conclusions:**

SGH’s experience demonstrates that embedding HB-HTA requires deliberate organizational design, leadership commitment, and stakeholder engagement. By combining concise narrative proposals with independent in-house assessments, hospitals can strengthen governance, foster accountability, and support value-driven innovation. This model offers a practical roadmap for institutions seeking to formalize HB-HTA within their decision-making processes.

## Introduction

Hospitals are often at the forefront of adopting new health technologies, making hospital-based health technology assessments (HB-HTAs) critical in informing whether, when, and how healthcare innovations should be taken up. This need for structured institutional decision-making is itself part of the broader HB-HTA landscape globally, as many health systems face pressure to innovate while dealing with rising costs. While the European Union (EU)-funded “Adopting Hospital-Based Health Technology Assessment in the EU” (AdHopHTA) project offered valuable recommendations for implementing HB-HTA (1;2), the real-world establishment of HB-HTA units (particularly outside Europe) remains limited and variably described.

International studies highlight persistent implementation gaps, including insufficient institutional recognition, limited dissemination of HB-HTA activities, unclear positioning within organizational structures, and inadequate resources to sustain the work (3–5). Despite these challenges, HB-HTA has gained traction worldwide and is increasingly used to support decision-making at the managerial level. It is most commonly implemented in large hospitals (>500 beds) and often operates in collaboration with national or regional HTA agencies (6). Across these contexts, HB-HTA is heterogeneous and dynamic, with no single best model; rather, its form must be adapted to the hospital’s priorities, governance structures, and internal workflows (2;4).

This global experience resonates with Singapore’s healthcare landscape, which simultaneously faces mounting pressures from rising costs, increasing service demands, and rapid technological advancement. Hospitals such as Singapore General Hospital (SGH) – the country’s flagship hospital – must balance innovation ambitions with fiscal discipline and rigorous evaluation. SGH operates 1,785 acute hospital beds – nearly one-quarter of the nation’s public acute beds. Supported by over 10,000 staff, SGH handled 128,000 emergency visits, 80,000 admissions, and 700,000 specialist clinic attendances in 2023 (7). Recognized as an important part of a leading Academic Medical Center (8), SGH partners with Duke-NUS Medical School and serves as the flagship of the SingHealth cluster, working collaboratively with its institutions to deliver comprehensive tertiary care.

Besides the aforementioned pressures, a confluence of factors further accelerated the adoption of HB-HTA at SGH. Following the coronavirus disease 2019 pandemic, the hospital grew increasingly cognizant of its rising healthcare expenditures. Concurrently, clinicians expressed heightened interest in adopting emerging technologies, particularly in pharmaceuticals, artificial intelligence, and robotics. Recognizing that existing decision-making processes were insufficient to support such innovations, hospital leadership saw the need for a pragmatic, multipronged approach to embed HB-HTA into operational workflows.

Therefore, in this article, we describe SGH’s approach to institutionalizing HB-HTA through a pragmatic, context-specific model that addresses several of the implementation challenges identified in the international literature.

## The three-pronged strategy for establishing an HB-HTA framework

Previously, proposals for high-cost technologies and programs were submitted through static forms that lacked sufficient context and detail, limiting their credibility and usefulness. Without a dedicated HTA team, decisions were made by senior management based on incomplete or suboptimal information. Recognizing these limitations, the hospital’s executive leadership developed a three-pronged strategy to establish an HB-HTA framework. This approach comprised structured narrative proposals, the formation of a dedicated HB-HTA team, and training programs to institutionalize HB-HTA principles. The aim was to strengthen SGH’s capacity to critically evaluate and adopt impactful innovations while mitigating the risks of both wrongful commission (adopting unsuitable technologies) and wrongful omission (overlooking valuable opportunities). By embedding evidence-based evaluations into decision-making processes, SGH sought to improve operational efficiency, ensure strategic alignment, and foster sustainable innovation. Notably, the emphasis on strategic alignment reflects one of the differentiated characteristics of HB-HTA compared to HTAs at national or regional levels, as highlighted in the AdHopHTA handbook, which informed the design and implementation of our framework (1).

## Narrative proposals enable clarity and rigor in HB-HTAs

Inspired by Amazon’s six-pager memo (9;10), SGH developed a structured, two-page narrative proposal template. The narrative format promotes clearer thinking, strengthens strategic alignment, and encourages evidence-based reasoning on the part of the proposer (10). Writing in narrative form requires a deep understanding of the proposal’s objectives, assumptions, and execution plan. Compared to traditional formats such as static forms or slide decks, the narrative approach offers higher information density. It also requires proposers to explicitly link their ideas to organizational priorities and address key considerations for adoption and implementation.

SGH’s narrative proposal template consists of seven standardized sections: title, introduction, goals, current state and unmet needs, lessons learned, proposed solution with strategic implementation plan, and organizational impact (Table 1). The title should be clear, succinct, and descriptive, accurately conveying the proposed technology or program. The introduction sets the direction for the rest of the document, beginning with the aim of the proposal, supported by one or two key claims, and followed by relevant context. It should clearly define the problem statement(s) and briefly outline the proposed solution, whether a program or technology. This section is kept concise, limited to two paragraphs.

**Table 1.** The narrative proposal template

**1. Title**
Provide a clear, succinct and descriptive title that describes the new technology. *Example: Acquisition of a novel surgical robot for intra-vascular surgery in patients with severe and complex peripheral vascular disease (PVD).*
**2. Introduction**
Set the general direction of the proposal. State the aim of the proposal. Provide relevant contextual information. Detail the problem statement(s) and describe concisely the technological solution. *Example: Singapore has one of the highest rates of PVD in the world (citation). An estimated 1 in 10 persons over the age of 65 suffer from PVD (citation)… Currently, the gold standard treatment for PVD is … However, robotic surgery promises to … Acquiring this robot will allow SGH surgeons to perform specialized vascular surgery that will…*
**3. Goals**
List 3–5 goals in bullet points. Describe the metrics for success. Goals should be specific, measurable, achievable, relevant and time-bound (SMART goals). *Example: With this new technology, we aim to achieve the following goals:* *Launch a new service to treat a monthly average (number) of patients with severe and complex PVD by (date).* *Utilize the robot to conduct (number) research studies exploring advanced endovascular surgical techniques.*
**4. Current state and unmet needs**
Provide a snapshot of current data to inform the reader of the current activities/state and therefore define any unmet needs. *Example: Patients suffering from severe PVD typically have a drastic reduction in their quality of life… The incidence of patients with severe and complex PVD is on the rise, on average the vascular surgery department encounters (number) of such patients (cite internal data). Current surgical techniques involve …. However, these have shown to be not beneficial in patients with severe and complex PVD (citation). Recent advancements with endovascular robotic surgery has demonstrated clinical benefits (citation). Early evidence suggests that robotic surgery is potentially cost-effective for complex patients (citation) as it reduces the need for ….*
**5. Lessons learned**
Reflect on what happened in the past to drive a discussion of past successes and failures relevant to the overall topic and goals. Translate these past lessons into actionable insights to support this proposal. *Example: The vascular surgery department has experience with adopting novel robotic techniques. Previously the department adopted … Past lessons learnt through the adoption of that technology included the need for …*
**6. Proposed solution with strategic implementation plan**
Present information about safety, clinical-effectiveness and cost-effectiveness of the proposed solution clearly. Then, provide a narrative to describe how the technology will be implemented, consider using a logic model to explain the implementation process (if suitable). The narrative should be coherent with the goals stated. Provide justification on why the proposed programme or technology is expected to be superior to the current state or other comparable technologies. Provide pertinent details regarding the execution including financial and operations-related projections.
**7. Organizational Impact**
Discuss how the proposed technology will contribute to the departmental, divisional, and organizational strategic positioning and long-term competitive advantages.
**8. Appendix**
Literature references and pertinent information should be placed in the appendix as supporting information.

The goals section outlines the metrics for success, enabling evaluators to interpret the remainder of the document through a consistent lens. Proposers are encouraged to present three to five goals that adhere to the SMART criteria (specific, measurable, achievable, relevant, and time-bound) (11).

The section on current state and unmet needs justifies the problem statement with relevant operational data and defines the gaps the proposal seeks to address. Proposers are also required to reflect on prior lessons learned, drawing on past successes and failures relevant to the technology’s implementation, and translating these into actionable insights.

In the proposed solution with a strategic implementation plan, proposers should describe the technological solution by presenting evidence of its safety, clinical and cost-effectiveness, and outlining how it will be implemented – ideally using a logic model to illustrate the process. The narrative should align with the stated goals and provide a clear rationale for why the proposed program or technology is expected to outperform current solutions or alternatives, thereby addressing the identified unmet needs. This section should also include key execution details, such as financial and operational projections. The organizational impact section then explains how the proposal will contribute to the departmental, divisional, and institutional strategic positioning, as well as to long-term competitive advantage.

To ensure brevity and accessibility for busy senior leaders, proposals are limited to two pages. However, proposers may include supplementary material in an appendix, for which there is no page limit. This approach prioritizes the most critical top-line information while ensuring that detailed supporting evidence remains readily available for review. While information on clinical and cost-effectiveness can often be sourced from reputable academic or industry publications, context-specific details – such as the local problem statement, operational realities, unmet needs, and the strategic plan – must be derived from local sources. The narrative proposal, therefore, plays an essential role in capturing this information, enabling well-rounded and contextually relevant decision-making.

## Establishing a dedicated team to impart HB-HTA principles and conduct assessments

To support the operationalization of HB-HTA at SGH, a dedicated team – referred to internally as the Impact Assessment, Program Evaluation, and Implementation Research (IEI) team – was established within the hospital’s Health Services Research Unit (12). The eight-member team comprises of public health physicians and health services researchers with expertise in impact assessment, economic evaluation, program evaluation, and implementation science. It plays a dual role: providing training to proposers and conducting independent evaluations.

As both the two-page narrative proposal format and HB-HTA were new to the wider hospital, it was essential to engage and train stakeholders. In the lead-up to the annual proposal call (internally referred to as “The Workplan”), the IEI team adopted a flipped classroom approach, complemented by a half-day interactive workshop. This format allowed participants to review foundational materials in advance and engage more deeply during the session. Training content covered the purpose and process of HB-HTA, techniques for developing high-quality proposals, the creation of logic models to strengthen implementation planning, and the effective use of the two-page proposal template.

Most HB-HTAs are conducted during SGH’s annual workplan cycle, which begins in September, although ad-hoc assessments are occasionally performed outside this period. During the workplan, senior management reviews numerous proposals with varying resource requirements. To prioritize those with the greatest potential impact, only proposals involving high costs – defined as exceeding USD $200,000 in capital expenditure or total cost – and pertaining to health technologies (including drugs, medical equipment, devices, diagnostic tests, and digital health technologies applied to specific medical services) are required to use the two-page narrative template and undergo assessment by the IEI team. Over a 2-week period, the IEI team conducts mini-HTAs of these proposals, reviewing the narrative submission, performing targeted literature searches to evaluate clinical and cost-effectiveness, and assessing organizational, political, and strategic implications. Their findings are consolidated into an impact assessment report, which is reviewed alongside the two-page proposal by the workplan committee, the Chairman Medical Board (CMB), divisional chairpersons, and other relevant stakeholders during the deliberation process. The impact assessment report is shared with proposers at the conclusion of the evaluation.

The workplan process can result in three broad outcomes. First, the committee may reject a proposal if evidence for clinical or cost-effectiveness is insufficient or if the strategic plan is inadequately developed – an outcome more common for nascent or untested technologies. Second, the committee may approve the proposal when it clearly addresses an important problem, presents a compelling strategic plan, and demonstrates a thorough understanding of the problem, the technology, and its implementation. Third, the committee may defer a decision, requesting that the IEI team conduct a more comprehensive HTA and collaborate with proposers to refine and strengthen the proposal before reconsideration.

## Lesson learnt from adopting HB-HTA: Process and hurdles

The implementation of HB-HTA at SGH was phased, beginning with a small-scale pilot during the 2023 work plan cycle, followed by full implementation in 2024. This staged approach was essential for introducing new processes, such as the two-page narrative proposal and formal HTAs, to stakeholders. Guided by principles of continuous improvement and organizational learning, insights from the pilot shaped the final rollout.

During the pilot, the team noted that some departments found the transition to the narrative proposal format challenging, particularly those with limited administrative support or unfamiliarity with HB-HTA requirements. In response, the HB-HTA team developed targeted training workshops to prepare proposers for the 2024 implementation.

## Early results and organisational learning

Between 2023 and 2024, 15 proposals, collectively exceeding USD $40 million in purchasing decisions, were submitted and evaluated. Five involved artificial intelligence applications for risk stratification, diagnosis, and resource optimization across multiple clinical disciplines. Four related to surgical robots and other therapeutic devices, three concerned diagnostic radiology equipment, and the remainder focused on patient monitoring devices. Specific product names and details are not disclosed to protect commercially sensitive information.

Following the formal rollout in 2024, the HB-HTA team conducted a formative evaluation involving proposers who had completed the training, as well as senior leaders engaged in the workplan process. Across 2024 and 2025, the training was completed by 31 and 24 participants, respectively. Engagement was quantified using the Net Promoter Score (13), yielding values of 52.4 percent in 2024 (21/31 respondents) and 50.0 percent in 2025 (24/24 respondents). These scores indicate a high likelihood of participants recommending the program to colleagues, reflecting both satisfaction and perceived value. Qualitative feedback further reinforced these findings, with participants describing the training as offering “a clear structure for drafting work plans, with practical insights for better project presentation and strategic planning” (2024) and “a comprehensive course with thoughtful pre-reading materials… a good foundational course, though more personal practice is needed to fully acquire the skill” (2025).

In 2024, the IEI team conducted a survey and targeted interviews among the senior management of SGH, designed to determine the perceived usefulness of the HB-HTAs to the leadership of the hospital. The surveys were completed by 11 individuals who were either divisional chairpersons or senior directors of relevant offices, as well as the CMB. The surveys demonstrated overwhelmingly positive feedback, with 10/11 (90.9 percent) respondents indicating that they were either “Satisfied” or “Very Satisfied” with the new processes. Moreover, survey respondents reported that both the narrative proposal template and HTAs significantly enhanced their confidence in decision-making compared to the previous format.

Two in-depth interviews were conducted with senior leaders from within this group. These offered more detailed and, thus, additionally valuable insights into the key benefits of the new processes from their perspective. The interviewees both highlighted that the narrative proposal template fostered greater consistency by requiring proposers to present contextual information cohesively across the various sections. The proposal template also facilitated more objective assessments by reducing the influence of subjective factors often associated with presentation pitches, encouraging decisions based on substance rather than style. Additionally, HB-HTA served as a crucial step to validate the data presented in the proposal and assess alignment to organizational priorities.

The narrative proposal also proved valuable in aligning proposals with SGH’s organizational strategy, especially the hospital’s “Peaks of Excellence” and research priorities (14). Consequently, there is an interest within the hospital’s executive leadership to explore how the two-page proposal template can be utilized more widely across the organization.

## Challenges and next steps

While the new processes were well received by senior management, additional areas for improvement were identified. A few stakeholders suggested that proposals could more effectively articulate the cost-effectiveness of proposed technologies or programs and their strategic benefits. They also noted that the quality of proposals could be enhanced to ensure that all pertinent details are presented clearly or appropriately referenced in the appendix. Looking ahead, demonstrating the value of these new processes to middle managers will be crucial. This effort is likely to be a multiyear process, requiring ongoing engagement with stakeholders to increase buy-in and build skills required for crafting high-quality narrative proposals.

Finally, while IEI’s impact assessment has proven valuable during the workplan process, there remains untapped potential to expand its application across other elements of decision-making. For example, greater synergy between the IEI team and proposers could enhance the overall process, provided the independence of the IEI team’s assessment is preserved. The IEI team could take on dual roles: acting as an enabler by conducting training and offering strategic consultations for proposers, and as an assessor delivering independent assessments to senior management.

## Considerations for implementation in other institutions

**Figure 1.**
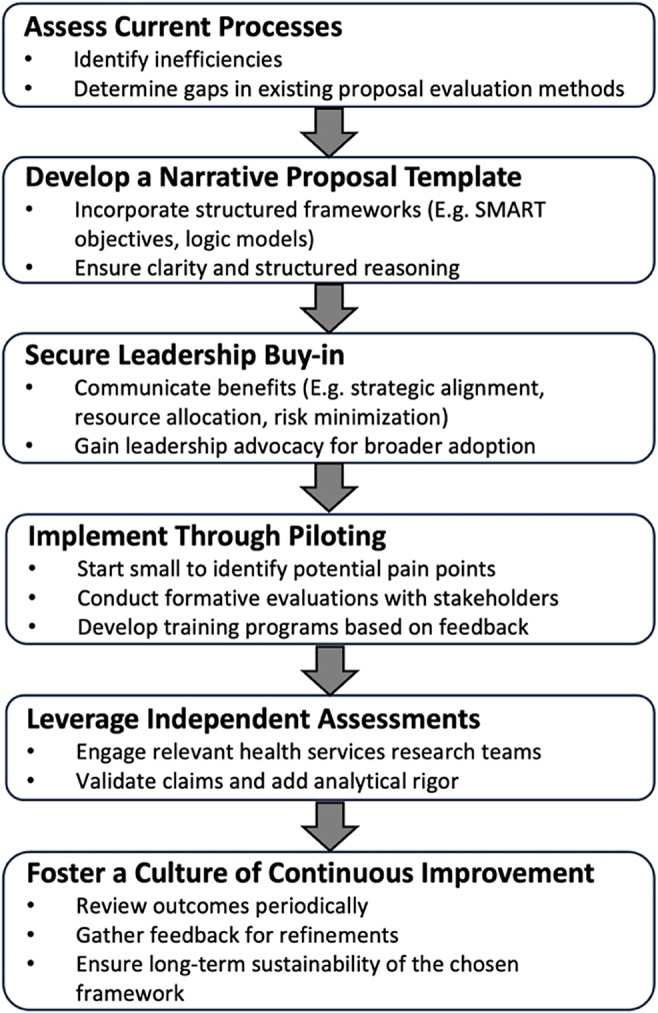
A six-step process for implementing the narrative proposal in hospitals.

Institutions seeking to establish an HB-HTA framework modeled on SGH’s experience may consider the six-step process outlined in Figure 1. The starting point is a critical appraisal of existing processes – specifically, identifying inefficiencies such as proposals lacking context, strategic alignment, or actionable insight. Recognizing these gaps is essential for designing a decision-making system that is responsive to organizational needs.

A tailored narrative proposal template is foundational to this effort. SGH’s two-page format offers a practical example, integrating tools, such as SMART objectives and logic models, to guide proposers in articulating their rationale, implementation plan, and anticipated outcomes. This format promotes structured thinking and ensures alignment with institutional priorities, while also enabling busy decision-makers to review proposals efficiently.

Securing early and explicit buy-in from senior leadership is critical. Leaders play a pivotal role in endorsing the value of a structured, evidence-informed approach – framing it as a means to enhance resource stewardship, reduce decision-making risk, and advance strategic coherence. Their support signals organizational commitment and encourages wider adoption.

Phased implementation improves the likelihood of success. Piloting the framework on a limited scale enables teams to detect friction points and refine the approach based on real-world feedback. At SGH, the initial pilot cycle informed the design of training workshops and support tools tailored to the needs of proposers. Early adopters – who experienced first-hand the benefits of the narrative proposal format – emerged as powerful advocates, helping to build momentum across departments.

Establishing a dedicated HB-HTA team is another key enabler. At SGH, this team provides both capacity-building and independent evaluations of high-cost proposals. Their expertise in public health, program evaluation, and impact assessment lends critical rigor to the review process, enhancing the quality of information available to leadership and supporting more accountable, data-driven decisions.

Finally, embedding a culture of continuous improvement ensures long-term sustainability. Routine evaluations, feedback mechanisms, and iterative refinement of the framework help maintain relevance and responsiveness to evolving institutional needs. Taken together, these steps provide a practical roadmap for hospitals aiming to strengthen innovation governance, institutionalize HB-HTA, and align new investments with strategic priorities.

## Conclusion

This experience at SGH demonstrates that institutionalizing HB-HTA demands deliberate attention to organizational design, leadership engagement, and cultural change. By combining a concise narrative proposal format with independent, in-house impact assessments, SGH has enhanced the quality, consistency, and strategic alignment of high-stakes decisions. Importantly, the approach empowers leaders to make better-informed choices while fostering a culture of accountability and continuous learning. As healthcare systems globally face mounting fiscal pressures and rapid technological advancement, SGH’s model offers a practical, scalable pathway for hospitals seeking to embed HTA principles into routine governance and build lasting institutional capacity for value-driven innovation.
